# Oxidative Stress Drives Cell Cycle Stalling, Apoptosis and Metabolic Suppression in Cystatin B Deficient EPM1 Patient iPSCs

**DOI:** 10.1111/cpr.70232

**Published:** 2026-05-19

**Authors:** Shekhar Singh, Lidiia Plotnikova, Teemu Tiukuvaara, Reetta Kälviäinen, Riikka H. Hämäläinen

**Affiliations:** ^1^ A.I. Virtanen Institute for Molecular Sciences, University of Eastern Finland Kuopio Finland; ^2^ Epilepsy Center, Kuopio University Hospital, Member of the European Reference Network, EpiCARE Kuopio Finland; ^3^ School of Medicine, Faculty of Health Sciences Institute of Clinical Medicine, University of Eastern Finland Kuopio Finland

**Keywords:** apoptosis, cell cycles, cystatin B, DNA damage, EPM1, metabolism, proliferation

## Abstract

Cystatin B (CSTB) is an inhibitor of cysteine proteases, particularly cathepsins. Biallelic loss‐of‐function mutations in the *CSTB* gene are causative for progressive myoclonic epilepsy type 1 (EPM1), a neurodegenerative disorder characterised by stimulus‐sensitive myoclonus and generalised tonic–clonic seizures. Pathomechanisms underlying the disease progression include disturbed proteostasis, increased oxidative stress, neuroinflammation and increased neuronal apoptosis. CSTB downregulation can also lead to cell cycle defects and reduced cell proliferation. Conversely, overexpression of CSTB has been reported to drive proliferation and survival of cancer cells, emphasising the important role of CSTB in cell growth and survival. In the current study, we focused on the role of CSTB in regulating proliferation and survival of EPM1 patients' stem cells. We reprogrammed EPM1 patient fibroblasts into induced pluripotent stem cells (iPSCs), a highly proliferative cell type. Patient cells manifested poor growth and increased apoptosis. Further, EPM1 cells showed oxidative stress, increased lysosomal activity, increased DNA damage and suppressed metabolism. Treatment with antioxidants rescued the growth phenotype and activated metabolism, suggesting oxidative stress as the cause and suppressed metabolism as a protective response. Our data strengthen the central role of CSTB in supporting cell survival and growth. Elucidating the regulatory role of CSTB expression in cell growth can advance our understanding of the pathophysiological mechanisms underlying both EPM1 and cancer and could inform the development of novel therapeutic strategies.

## Graphical abstract

CSTB deficient EPM1 iPS cells manifest increased lysosomal activity and oxidative stress, which lead to DNA damage, cell cycle defects and increased apoptosis. As a protective response, metabolism is suppressed. Image created by BioRender https://BioRender.com/t44oc6h.

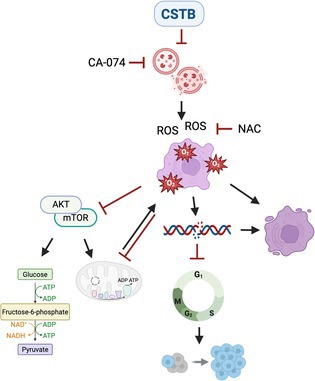

## 1 Introduction

Cystatin B (CSTB) is an intracellular inhibitor of thiol‐dependent proteases, particularly cathepsins. It plays an important role in inhibiting protein degradation but has also additional roles beyond proteostasis. It influences cell cycle progression, cell proliferation and mitochondrial function and protects cells from oxidative stress and DNA damage [1, 2, 3, 4, 5, 6]. CSTB can localise in the cytoplasm, nucleus, lysosomes or mitochondria [1], and it can be secreted into the extracellular environment, suggesting roles that extend beyond intracellular functions [7].

Bi‐allelic loss‐of‐function mutations in the *CSTB* gene lead to progressive myoclonic epilepsy type 1 (EPM1), a rare neurological condition also known as Unverricht–Lundborg disease (ULD) [8]. EPM1 patients commonly experience light‐sensitive seizures, stimulus‐induced myoclonus and motor incoordination [1, 8, 9]. The majority of the EPM1 patients have biallelic dodecamer repeat expansion at the promoter region of the *CSTB* gene, a hypomorphic allele that leads to reduced expression of the CSTB protein. A minority of the patients are compound heterozygotes, with one copy of the repeat expansion variant and one point mutation in the *CSTB* gene. The disease‐associated point mutations also cause loss of function and usually result in a more severe clinical phenotype. The disease severity seems to inversely correlate with the amount of residual functional CSTB.

Biallelic complete null mutations of the *CSTB* gene lead to a phenotype of developmental encephalopathy. These patients present with microcephaly, early developmental delay and severe dyskinesia [10, 11]. In mice, Cstb knockdown leads to early onset seizures, progressive neurodegeneration and neuronal apoptosis, mimicking the EPM1 patient phenotype relatively well, despite complete depletion of the Cstb protein [12]. Mitochondrial dysfunction and increased oxidative stress have been detected in neuronal cells of *Cstb*‐mutant mice and are currently considered as underlying mechanisms for the neuronal loss [5, 10, 11, 12].

On the other side, overexpression of Cystatin B has been reported in several cancer types, where CSTB upregulation has been linked to increased proliferation, chemoresistance, invasion, metastasis and resistance to apoptosis [13, 14, 15, 16]. Thus, the significant role for Cystatin B in regulating cell proliferation and survival is evident.

In the current study, we generated induced pluripotent stem cells (iPSCs) from EPM1 patient fibroblasts. Here we report that in iPSCs, reduced CSTB expression leads to increased lysosomal activity and oxidative stress, which drive suppression of metabolism, reduced proliferation, increased DNA damage, cell cycle defects and increased apoptosis.

## 2 Materials and Methods

### 2.1 Patients and Cells

Three patients from Kuopio Epilepsy Center were involved in the study. All three present with a severe form of EPM1. Patients 2 and 3 carried a homozygous expansion mutation, while patient 1 was compound heterozygous for the expansion mutation and a c.202C > T (R68X) point mutation. IPSCs were generated from dermal fibroblasts using the Cytotune‐iPS 2.0 Sendai reprogramming kit (Invitrogen, A16517) and grown on Matrigel (Corning, 356231) coated plates in E8 medium (Gibco, A1517001) at 37°C in 5% CO_2_. Karyotyping (20 metaphases) was performed by G‐banding at AmbLab, Barcelona, Spain. TAKARA Cellartis lines (Ctrl‐1: ChiPSC7, Y00270; Ctrl‐2: ChiPSC18, Y00300; and Ctrl‐3: ChiPSC22, Y30010) were used as controls.

### 2.2 Generation of Transgenic Control Cell Lines

To generate additional control lines, plasmids were designed by the VectorBuilder platform to overexpress (pPB[Exp]‐Neo‐EF1A > hCSTB[NM_000100.4]) and silence (pPB[shRNA]‐Neo‐U6 > hCSTB[shRNA#1]) CSTB expression (Figure S1). Plasmid DNA was extracted by PureLink HiPure Plasmid Midiprep Kit (Invitrogen, K210004). Ctrl‐1 cells (1 × 10^6^) were transfected with 1 ug pPB[shRNA]‐Neo‐U6 > hCSTB[shRNA#1] and P‐3 cells UEFi004‐A line [17] in European hPSC registry, www.hpscreg.eu (1 × 10^6^), with 1 ug pPB[Exp]‐Neo‐EF1A > hCSTB[NM_000100.4], both together with 1 ug pCy43 plasmid [18] using Neon Transfection System (Invitrogen, MPK10096, 800 V, 20 ms, 2 pulses). Selection with G418 (Cayman Chemical, 13884) started 48 h after transfection, concentration gradually increasing from 100 to 200 μg/mL. Colonies were picked manually 5 days after transfection.

### 2.3 Cell Culture

The iPS cells were maintained in Essential 8 Medium supplemented with 1% Penicillin/Streptomycin on Matrigel‐coated dishes. For passaging, the cells were treated with 0.5 mM EDTA for 3–5 min, before collection and transfer to fresh Matrigel dishes. During passaging, the medium was supplemented with 10 μM Rock inhibitor (Selleckchem, Y‐27632).

### 2.4 Mutation Detection

To extract genomic DNA, cells were lysed with DNA lysis buffer (100 mM Tris–HCl (pH 8.5), 5 mM EDTA, 0.2% SDS, 200 mM NaCl) and Proteinase K (100 μg/mL, Thermo Scientific, EO0491) at 55°C overnight. DNA was precipitated with isopropanol, washed twice with 70% ethanol and resuspended in nuclease‐free water. Concentration was measured with NanoDrop ND‐1000 Spectrophotometer.

The presence of the expansion mutation was verified by PCR across the CSTB promoter region as reported earlier [17]. To amplify the point mutation in P‐3, the region was amplified (primers in Table S1), PCR product was purified with QIAquick PCR Purification Kit (Qiagen, 28104) and sequenced at Macrogen Europe.

### 2.5 Proliferation Assay

Cells were seeded on 24‐well plates at a density of 1 × 10^4^ cells/well (day 0) and cultured for three days. Cells were harvested by EDTA, resuspended in PBS and the cell number was counted daily using a haemocytometer.

### 2.6 MTT Assay

Cells were seeded on 96‐well plates at a density of 5 × 10^3^ cells/well. On day 3, 20 μL of MTT solution (5 mg/mL in PBS; Thermo, 158990010) was added and incubated for 4 h at 37°C. The medium was removed, and 150 μL of dimethyl sulfoxide (DMSO; Sigma‐Aldrich, D2650) was added to dissolve the formazan crystals. Absorbance was measured at 544 nm using a microplate reader Victor2 1420 multilabel counter (Wallac, Turku, Finland).

### 2.7 Cell Cycle Analysis

Cells were harvested, washed twice with cold PBS and fixed in 70% ethanol at −20°C. Fixed cells were centrifuged at 500 × g for 5 min, washed with PBS, resuspended in PBS containing 100 μg/mL RNase A (Thermo, EN0531) and incubated for 30 min at room temperature (RT). Propidium iodide (PI; Cayman Chemical, 10008351, 50 μg/mL final concentration) was added and samples were incubated for 10 min at RT. Cell cycle analysis was performed using a CytoFLEX S Analyser (Beckman Coulter, Brea, CA, USA, 50,000 events per sample). Data were analysed with FCS Express 6 software cell cycle distribution programme.

### 2.8 Apoptosis Assay

Apoptosis was assessed using the Annexin V‐FITC Apoptosis Detection Kit (Abcam, ab14085) according to the manufacturer's instructions. Samples were analysed immediately using a CytoFLEX S Analyser (Beckman Coulter, USA). Data was processed with FCS Express 6 software (De Novo Software, Pasadena, CA, USA).

### 2.9 Immunofluorescence

The cells were grown on coverslips, fixed with 4% paraformaldehyde (Sigma‐Aldrich) for 20 min at RT, and washed 3 times. Permeabilisation and blocking was with 0.1% Triton X‐100 and 10% horse serum in PBS for 1 h at RT. The samples were incubated with primary antibodies (Table S2) overnight at 4°C in PBS with 0.1% Triton X‐100 and 1% horse serum, washed twice with PBST and stained with secondary antibodies for 1 h at RT. The nuclei were stained with DAPI (1 μg/mL) and mounted with Fluoromount‐G (Southern Biotech; 0100–01). Imaging was done using a Zeiss Axio Observer Z1 microscope. γ‐H2A.X dots per nuclei (≈100 nuclei per group) were counted using Fiji software (ImageJ).

### 2.10 RT‐qPCR

Total RNA was isolated using Tritidyl G (VWR, A4051.0100). The cDNA was synthesised using total RNA, dNTPs (Thermo, R0192), random hexamer primers (Thermo, SO142) and Maxima reverse transcriptase in the presence of ribonuclease inhibitor (Thermo, EO0381). RT‐qPCR was run using Power SYBR Green PCR Master Mix (Thermo, 4367659) and StepOnePlus Real‐Time PCR System (ThermoFisher Scientific, USA). Primers are listed in Table S1. The relative expression levels were calculated with the ΔΔ Ct method and normalised against Beta‐Actin.

### 2.11 Western Blotting

Proteins were extracted using RIPA Lysis and Extraction Buffer (Thermo, 89901) supplemented with protease and phosphatase inhibitors (Thermo, A32966 and A32958). Protein quantification was done using Pierce BCA Protein Assay Kits (Thermo Scientific, 23225) with the Perkin Elmer Wallac 1420 Victor2 Microplate Reader at 544 nm. SDS‐PAGE gels were run at 100 V for 90 min, followed by wet transfer on PDVF membranes. The membranes were blocked for 1 h in 5% BSA, incubated overnight at 4°C with primary antibodies (Table S2), followed by 3 washes and incubation with secondary antibodies for 1 h at RT. For detection, Pierce ECL Western Blotting Substrate (Thermo Scientific, 32109) and ChemiDoc MP imaging system (Bio‐Rad, Hercules, CA, USA) were used. Densitometric analysis was done using Fiji (ImageJ) software.

### 2.12 Seahorse Assay

Oxygen consumption (OCR) and extracellular acidification (ECAR) were assessed with XF96 Extracellular Flux Analyser (Seahorse Bioscience, North Billerica, MA, USA). Cells were seeded at a density of 5 × 10^3^ cells per well, 2 days before the run. On the day of the run, the cells were washed with assay medium and incubated for 1 h in assay medium (including glucose, pyruvate and glutamine) at +37°C without CO2. After baseline measurements, a final concentration of 1 μM oligomycin (an ATP synthase inhibitor, Sigma‐Aldrich), 1 μM carbonyl cyanide p‐[trifluoromethoxy]‐phenylhydrazone (FCCP, a mitochondrial uncoupler, Sigma‐Aldrich) and 1 μM antimycin A (complex III inhibitor, Sigma‐Aldrich) together with 1 μM rotenone (complex I inhibitor, Sigma‐Aldrich) were added sequentially. The data were normalised to total protein content analysed with Pierce BCA Protein Assay (Thermo, 23225). The results were analysed with the Wave programme (Agilent Technology, Santa Clara, CA, USA).

### 2.13 Mitochondrial Content

For analysing mitochondrial mass and membrane potential, cells were stained with 20 nM Tetramethylrhodamine Methyl Ester Perchlorate (TMRM, Thermo Scientific, Waltham, MA, USA) or 200 nM MitoTracker Green (Invitrogen, M7514) for 10 min at +37°C. Analysis was performed using a CytoFLEX S Analyser (Beckman Coulter, Brea, CA, USA), acquiring 20,000 events per sample. Data were analysed with FCS Express 6 software.

### 2.14 CellRox and MitoSOX Assays

For ROS (reactive oxygen species) detection, cells were incubated with 5 μM CellROX Green Reagent (Thermo, C10444) for 30 min at 37°C. For mitochondrial superoxide detection, cells were incubated with 5 μM MitoSOX Red Mitochondrial Superoxide Indicator (Thermo, M36006) for 10 min at 37°C. Before collection with PBS, the cells were washed twice with warm PBS. Fluorescence was measured using a CytoFLEX S Analyser (Beckman Coulter, USA). Data were analysed with FCS Express 6 software.

### 2.15 Cathepsin B Activity Assay

Cells were harvested (5 × 10^6^ cells per condition), washed with ice‐cold PBS and lysed in 50 μL chilled Cathepsin B Cell Lysis Buffer (Abcam, ab65300). Lysates were incubated on ice for 10–30 min, then clarified by centrifugation at top speed for 2–5 min at 4°C. Supernatants were collected and kept on ice until use. For the activity reaction, 50 μL of lysate (or 50–200 μg protein adjusted to 50 μL with lysis buffer) was added to a black 96‐well plate in duplicate. 50 μL Cathepsin B Reaction Buffer was added, followed by 2 μL of 10 mM Ac‐RR‐AFC substrate (final concentration 200 μM). Plates were incubated at 37°C for 2 h protected from light, and fluorescence was measured at Ex/Em 400/505 nm. Fluorescence background was subtracted, and Cathepsin B activity is reported as relative fluorescence.

### 2.16 Lysosomal Activity Assay

For inhibitor control, cells were treated with 1 × Cytochalasin D under basic conditions (Abcam, ab234622). Self‐Quenched Substrate was reconstituted according to the manufacturer's instructions and added to cells (7.5 μL substrate per 500 μL medium). Cells were incubated for 1 h at 37°C, collected on ice, washed twice with ice‐cold Buffer I and resuspended in 1 mL 1 × PBS for acquisition. Flow cytometry was performed on a CytoFLEX using 488 nm excitation. The lysosomal intracellular activity was quantified as mean fluorescence intensity (MFI). 50,000 total cellular events were acquired per sample.

### 2.17 Treatments

The cells were treated with 200 μM N‐acetyl‐L‐cysteine (NAC, Sigma‐Aldrich, A9165) or 0.5 μM Cathepsin B Inhibitor III (Thermo, CA‐074 J65347) for 3 days and analysed as described earlier.

### 2.18 Statistical Analysis

The data were analysed using GraphPad Prism. Experiments were repeated at least three times. The data are presented as mean ± SD. For comparisons among > 2 groups, one‐way ANOVA was performed followed by Tukey's multiple‐comparisons test (all pairwise comparisons). For experiments with two independent variables, two‐way ANOVA was used to test main effects and interactions, followed by Sidak's multiple‐comparisons test for planned pairwise contrasts. Statistical significance: *p* < 0.05 (*), *p* < 0.005 (**), *p* < 0.0005 (***), *p* < 0.00005 (****).

## 3 Results

### 3.1 Generation of Patient‐Derived iPSCs

The patient iPSCs displayed typical pluripotent stem cell morphology (Figure S2A) and expressed key pluripotency markers, including Homeobox protein NANOG, Tumour‐Related Antigen 1–60 (TRA‐1–60), and Tumour‐Related Antigen 1–81 (TRA‐1–81), as confirmed by immunofluorescence staining (Figure S2B). The expression of additional pluripotency‐related genes, DNA (cytosine‐5)‐methyltransferase 3 beta (*DNMT3B*), *NANOG*, and Sex determining region Y‐box 2 (*SOX2*), was validated with quantitative PCR (Figure S2C). The absence of residual Sendai virus vectors was confirmed (Figure S2D), and karyotype analysis revealed a normal chromosomal profile (Figure S2E).

### 3.2 *CSTB* Expression Is Downregulated in Patient iPSCs

**FIGURE 1:**
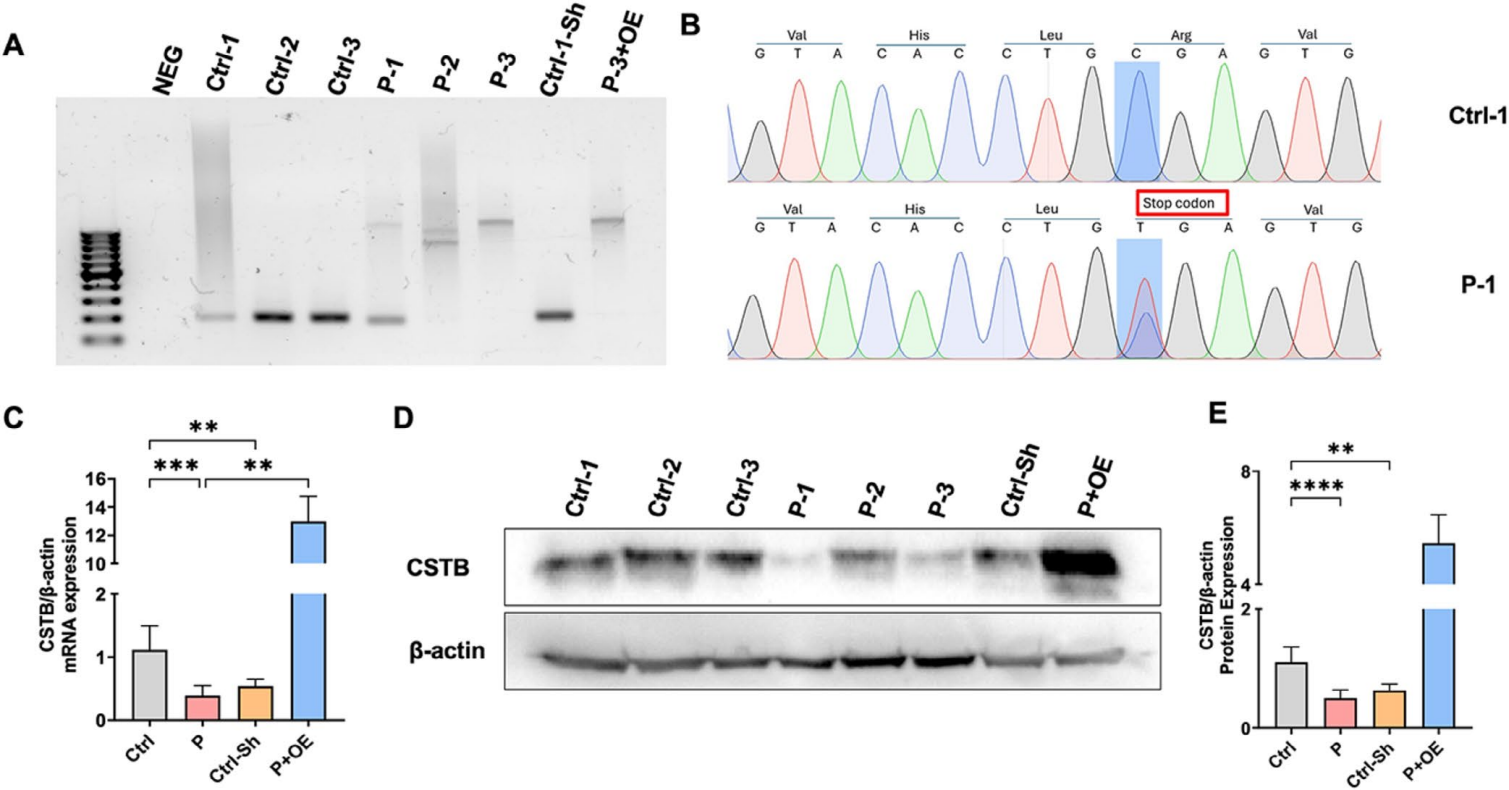
The expansion mutation leads to decreased CSTB expression in patient cells. (A) Genomic PCR over the *CSTB* promoter region. (B) Sanger sequencing of the c.202C > T(R68X) mutation in exon 3 of *CSTB* in P‐1. (C) q‐PCR of the CSTB mRNA expression. (D) Western blot and (E) quantification of the data for CSTB protein expression. Experiments (C, D) were repeated at least three times. Data are presented as mean ± SD. *p* < 0.05 (*), *p* < 0.005 (**), *p* < 0.0005 (***), *p* < 0.00005 (****).

PCR over the *CSTB* promoter region revealed that all three patients carried the dodecamer repeat expansion mutation with ~60–80 repeat copies (Figure 1A). Patients 2 and 3 were homozygous, while P‐1 carried one allele of the expansion mutation and Sanger sequencing verified the c.202C > T point mutation in the other allele (Figure 1B). Quantitative‐PCR and western blot assay revealed significant downregulation of both CSTB mRNA and protein expression in the patient lines (Figure 1C–E). Decreased CSTB expression was evident also in the Ctrl‐Sh line, whereas CSTB expression was drastically increased in the *P*+OE line (Figure 1C–E). These results verify the presence of the patient mutations in the iPSC lines and show that CSTB expression is downregulated in them, as well as verify a correct functional effect in each transgenic line.

### 3.3 CSTB Regulates Cell Proliferation, Cell Cycle and Apoptosis in iPSCs

**FIGURE 2:**
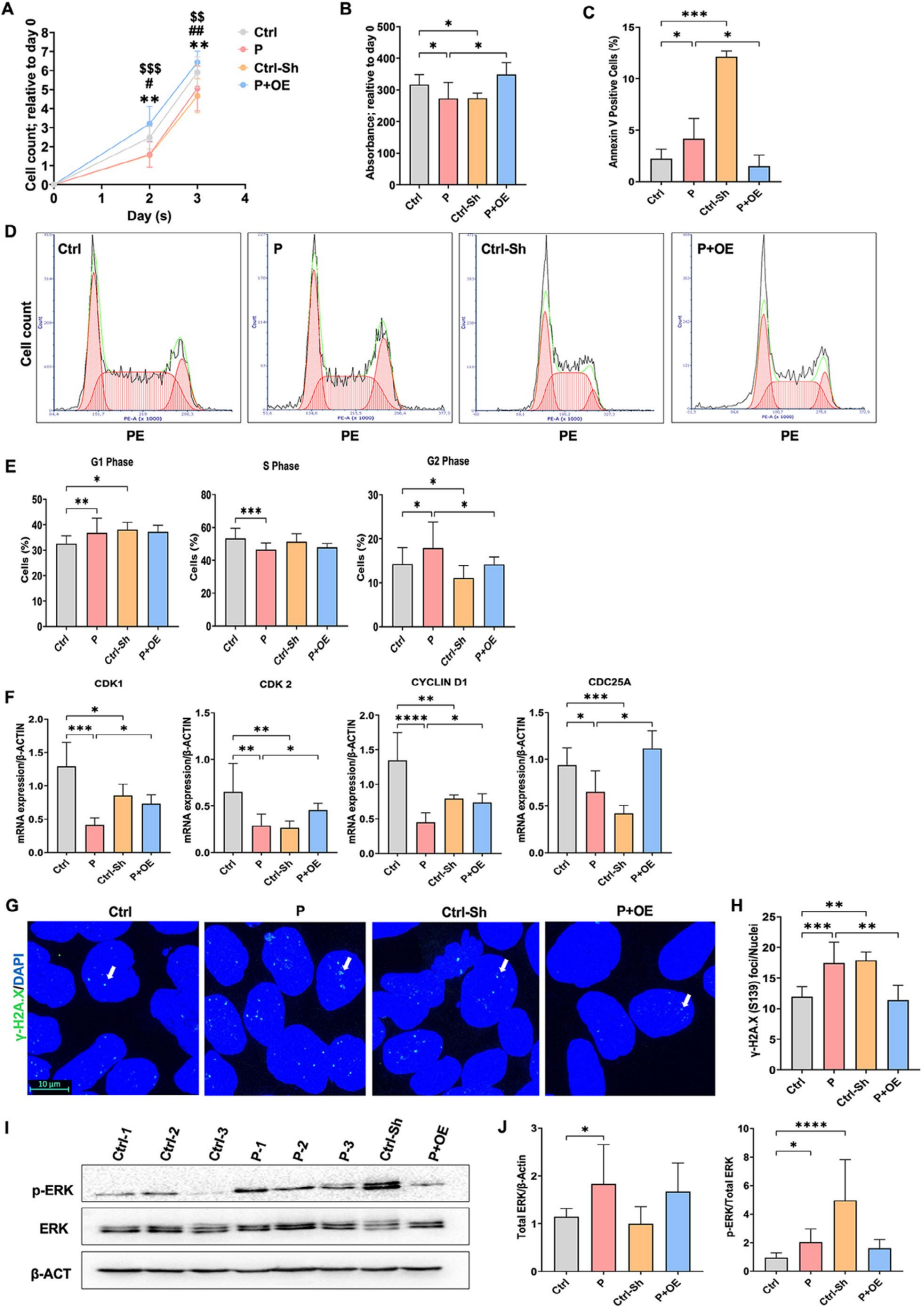
Reduced cell growth and increased apoptosis in EPM1 patient iPSCs are due to low CSTB expression. (A) Growth curve of the iPS cells over 3 days. Significance: P vs. ctrl**; Ctrl versus Ctrl‐Sh^##^; P versus *P*+OE^$$^. (B) MTT assay, *n* = 12 per cell line, 3 experimental repeats. (C) Analysis of apoptosis by Annexin V staining and flow cytometry. (D) Representative results from cell cycle analysis and (E) quantification of the proportion of cells in G1, S and G2 phases. (F) q‐PCR for cell cycle markers *CDK1, CDK2*, *CYCLIN D1*, *CDC25A.* (G) Immunocytometry (scale bars 10 μm) and (H) quantification of the γ‐H2A.X dots per nucleus (*n* = ≈100 nuclei per cell line). (I) Western blot and (J) quantification of total ERK and p‐ERK (Thr202/Try204). Experiments (A–F; I, J) were repeated at least three times. Data are presented as mean ± SD. *p* < 0.05 (*), *p* < 0.005 (**), *p* < 0.0005 (***), *p* < 0.00005 (****).

When establishing the iPSC lines, it became evident that the growth of the patient cells was significantly reduced. To quantify this, a proliferation assay was performed. A substantial decrease in cell proliferation was seen in patient cells when compared to Ctrl cells (Figure 2A). The control line with shRNA induced CSTB downregulation, mimicked the growth of patient lines, whereas overexpression of CSTB in a patient line increased cell proliferation above control cell proliferation (Figure 2A). MTT assay showed reduced formazan formation in patient cells, indicating decreased mitochondrial activity, and consistent with impaired cell growth (Figure 2B). We next analysed if cell death was increased in EPM1 patient iPSCs and flow cytometry analysis revealed a significantly higher number of Annexin V positive apoptotic cells in patient lines when compared to Ctrl lines (Figure 2C).

To investigate if also cell proliferation was altered, a cell cycle analysis was performed. A significant reduction in the proportion of cells in the S phase was detected in the patient cells when compared to Ctrl cells (Figure 2D,E). In addition, q‐PCR assay showed downregulation of key cell cycle genes including Cyclin‐dependent kinase 1 (*CDK1*), Cyclin‐dependent kinase 2 (*CDK2*), *CYCLIN D1* and Cell Division Cycle 25A (*CDC25A*) (Figure 2F).

CSTB has been suggested to protect cells against DNA damage [19], we thus next stained the cells with γ‐H2A.X(S139) antibody (phosphorylated histone variant H2AX). Quantification of γ‐H2A.X foci per nuclei showed a drastically increased number of DNA double strand breaks (DSBs) in the patient cells when compared to control cells (Figure 2G,H). To assess stress‐ and DNA damage–associated signalling pathways, we performed Western blot analysis of total ERK1/2 (Extracellular signal‐regulated kinase) and phosphorylated ERK1/2 (p‐ERK) and detected an increase in ERK phosphorylation accompanied by an increase in total ERK in patient cells (Figure 2I,J). The transgenic lines verified that these changes were due to the low CSTB expression (Figure 2A–J). These data show that CSTB deficiency leads to poor growth of the EPM1 iPSCs due to increased apoptosis, increased DNA damage and stalled cell cycles.

### 3.4 Low CSTB Levels Lead to Reduced Metabolism in EPM1 Patient iPSCs

**FIGURE 3:**
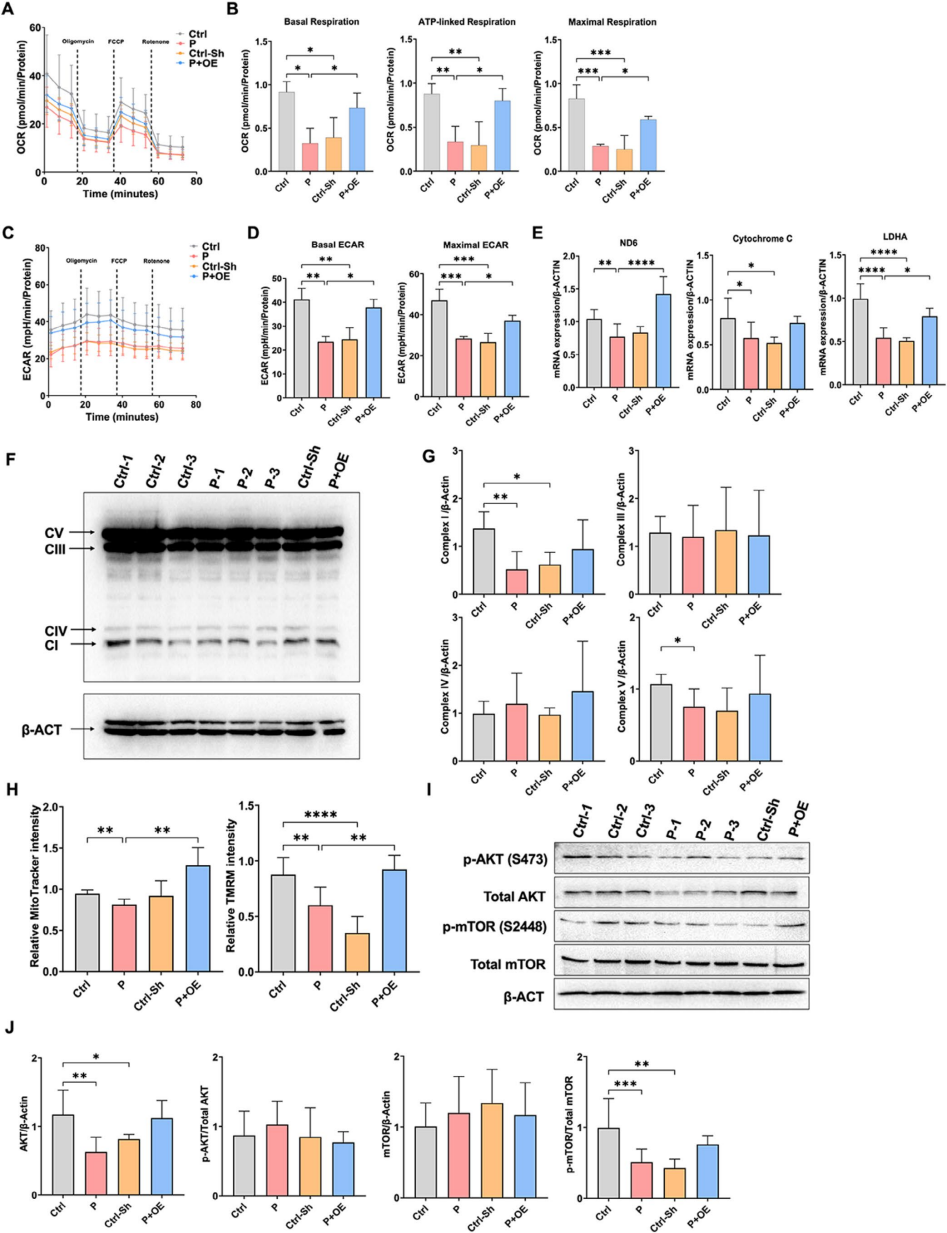
Metabolism is reduced in EPM1 patient iPSCs. (A‐D) Seahorse analysis of oxygen consumption and extracellular acidification. (A) OCR at basal level and after inhibition of the RC with oligomycin, FCCP and Antimycin A and Rotenone Data is normalised to total protein content. (B) Quantification of the OCR data Basal ATP linked and maximal respiration. (C) ECAR and (D) quantification of the ECAR data, basal and maximal ECAR. (E) q‐PCR analysis of genes involved in metabolic processes *ND6*, Cytochrome C and *LDHA*. (F) OXPHOS western blot and (G) quantification of Complexes I, III, IV and V. (H) Mitochondrial content, TMRM and Mito tracker staining on live cells and analysis by flow cytometry. (I) Western blot and (J) quantification of total AKT, p‐AKT (S473), total mTOR and p‐mTOR (S2448). All experiments were repeated at least three times. Data are presented as mean ± SD. *p* < 0.05 (*), *p* < 0.005 (**), *p* < 0.0005 (***), *p* < 0.00005 (****).

We next analysed cellular metabolism utilising the Seahorse XF‐96 Extracellular Flux Analyser and the mito stress test. Analysis of oxygen consumption and extracellular acidification, as a proxy for glycolysis, showed that both respiration and glycolysis were drastically reduced in patient cells (Figure 3A–D). The patient iPSCs showed low basal, ATP‐linked, and maximal respiration when compared to Ctrl cells (Figure 3B). Further, instead of compensating for the low respiratory activity with increased glycolysis, glycolytic activity was also low in patient cells, and both basal and maximal glycolysis were reduced (Figure 3D). In line with the suppressed metabolism, q‐PCR showed downregulation of metabolic genes including NADH dehydrogenase subunit 6 (*ND6*), Cytochrome C and Lactate Dehydrogenase A (*LDHA*) in the patient lines when compared to Ctrl cells (Figure 3E). The transgenic lines verified that these changes were due to altered CSTB expression (Figure 3A–E).

As the respiratory activity was so drastically reduced, we further analysed mitochondrial content of the cells. Western blot analysis using a total OXPHOS antibody cocktail revealed a reduction in Complex I and V protein abundance in CSTB patient iPSCs compared with controls (Figure 3F,G) and TMRM and Mito‐tracker green dyes for active and total mitochondrial staining, respectively, showed a reduction in active mitochondrial content (Figure 3H), whereas overexpression of CSTB led to increased mitochondrial mass (Figure 3H).

The AKT (Protein Kinase B)/mTOR (mechanistic target of rapamycin) pathway is a key regulator of cell metabolism [20, 21]. We thus further analysed AKT and mTOR activity by western blot. While the activation level i.e., Ser473 phosphorylation of AKT was unchanged in patient cells, the total AKT levels were significantly reduced when compared to Ctrl cells (Figure 3I,J), thus resulting also in low levels of the active form. In line with this, activation of mTOR (P‐Ser2448) was significantly reduced in patient cells (Figure 3I,J) and the transgenic lines verified that this was due to CSTB expression (Figure 3I,J).

Altogether, these data show that low CSTB expression reduces metabolic activity of iPSCs by downregulating both cellular respiration and glycolysis, likely through suppressed AKT/mTOR activity.

### 3.5 Low Cystatin B Expression Increases Lysosomal Activity and Non‐Mitochondrial ROS Production

**FIGURE 4:**
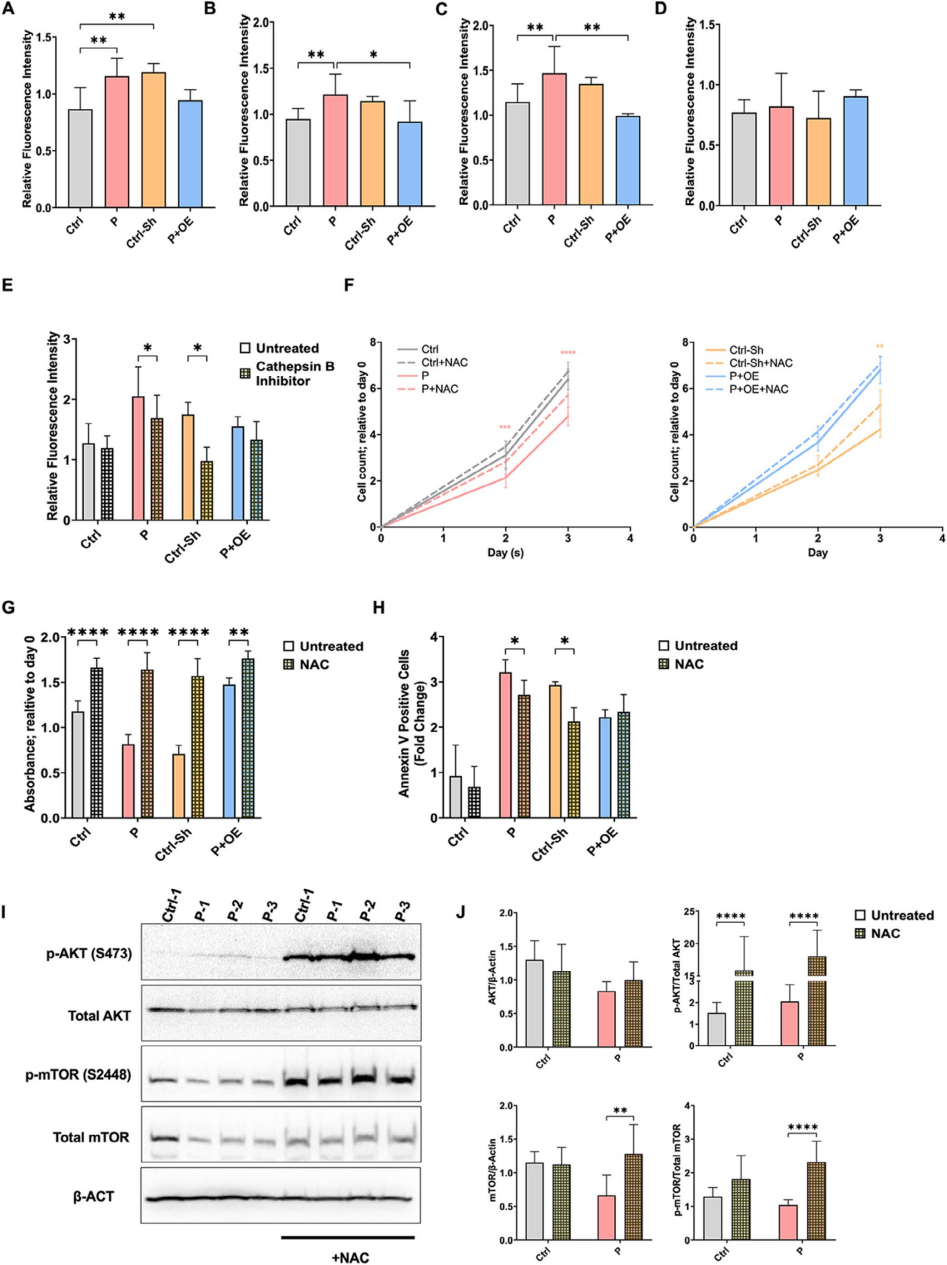
Oxidative stress is increased in patient iPSCs. (A) Cathepsin B activity assay. (B) Lysosomal activity assay. (C) CellROX assay. (D) MitoSOX assay. (E) CellROX assay after Cathepsin B inhibition. (F) Growth curves after NAC treatment. (G) MTT assay after NAC treatment. (H) Analysis of apoptosis by Annexin V staining and flow cytometry after NAC treatment. (I) Western blot and (J) quantification of total AKT, p‐AKT (S473), total mTOR and p‐mTOR (S2448) with and without NAC treatment. All experiments were repeated at least three times. Data are presented as mean ± SD. *p* < 0.05 (*), *p* < 0.005 (**), *p* < 0.0005 (***), *p* < 0.00005 (****).

As CSTB is an inhibitor of lysosomal cysteine proteases, we next evaluated lysosomal protease activity. Patient cells showed a significant increase in cathepsin B activity (Figure 4A) as well as elevated lysosomal intracellular activity (Figure 4B). CSTB defects have earlier been linked to oxidative stress. Thus, we analysed ROS levels, both in mitochondria and in cytoplasm. CellROX assay showed that cytoplasmic ROS levels were significantly increased in patient cells (Figure 4C). However, MitoSOX assay indicated that mitochondrial superoxide production was not increased (Figure 4D). To assess whether this intracellular ROS could be linked to the increased lysosomal activity, we further treated the cells with Cathepsin B inhibitor (CA‐074). While higher doses were detrimental for all lines (data not shown) a low CA‐074 concentration (0,5 μM) reduced ROS specifically in patient cells (Figure 4E).

These data show that the patient cells show increased cathepsin and lysosomal activity as well as increased non‐mitochondrial oxidative stress putatively arising from lysosomes.

### 3.6 NAC Rescues Proliferation, Reduces Apoptosis and Activates Metabolism in CSTB Patient iPSCs

To test whether oxidative stress contributes functionally to the reduced viability of patient cells, we treated cells with the antioxidant N‐acetylcysteine (NAC) and assessed growth, metabolic activity and apoptosis. NAC treatment significantly improved cell growth (Figure 4F), which was accompanied by a significant increase in MTT signal (Figure 4G), as well as significantly decreased apoptosis (Figure 4H). Further, both AKT and mTOR phosphorylation significantly increased in patient cells upon NAC treatment, indicating activation of these pathways and metabolism in treated cells (Figure 4I,J). These data show that oxidative stress drives the proliferation and survival defects in EPM1 iPSCs and suggests that the metabolic suppression is a protective response.

## 4 Discussion

In this study we investigated the mechanisms controlling poor growth and survival of CSTB deficient iPS cells. The role for CSTB in maintaining cellular proteostasis has been evident for years [1]. However, studies both on EPM1 patient cells and on cancer cells have suggested that CSTB has critical roles also in several other pathways, including controlling apoptosis and cell cycle and in DNA damage response [13, 14, 15, 16, 22]. Here, by utilising highly proliferative iPSCs with drastically reduced CSTB expression from EPM1 patients, we verify that these pathways are involved also in EPM1 pathology. Further, transgenic lines, with silencing of the CSTB in control cells and overexpression of CSTB in patient cells, confirm that these changes in EPM1 cells indeed arise from altered CSTB expression. The EPM1 disease severity seems to inversely correlate with the amount of residual functional CSTB and often patients with one null mutation tend to show a more severe phenotype than those homozygous for the expansion mutation. We did not see any significant difference in the severity of the cellular defects between our patients. However, our patients exhibited expansion mutations that were on the longer end of the reported size spectrum (around 60–80 copies) and we neither saw clear differences in CSTB expression among the patients. Larger patient material with more variation in the genetic background could reveal also variation in cellular responses.

Cell growth depends on two aspects, proliferation and cell death and our data shows that CSTB controls both in iPSCs. While the regulation may happen through multiple different pathways, the main driver for these defects in EPM1 iPSCs seems to be oxidative stress. Cytoplasmic ROS levels were significantly increased and antioxidant treatment both increased proliferation and reduced apoptosis in patient cells. Interestingly, but in line with the very low oxidative metabolism, mitochondrial ROS was not increased, suggesting that the ROS is primarily derived from non‐mitochondrial sources like cytoplasmic oxidases, lysosomes and/or peroxisomes [23]. A putative cause for the increased ROS in EPM1 is lysosomes, as low CSTB activity can increase cathepsin activity [24] and alter lysosomal function, and lysosomes can generate ROS through Fenton chemistry [25, 26]. The patient cells showed increased cathepsin B and lysosomal activities, and cathepsin B inhibition reduced cytoplasmic ROS specifically in patient cells, indicating that lysosomes may be the source for the excess ROS in EPM1 cells. CSTB has also been reported to have protective roles against oxidative stress [19, 27], the absence of which may further aggravate the phenotype.

The metabolism in EPM1 patient iPSCs was significantly suppressed. CSTB has been suggested to control mitochondria, and our data supports this, as low CSTB expression led to reduced active mitochondria and overexpression of CSTB increased mitochondrial content in cells. CSTB is not classified as a metabolic regulator, yet we saw decreased activation of the AKT/mTOR pathway, which could be the underlying mechanism for the suppressed metabolism in EPM1 patient cells. AKT and mTOR are central regulators of cellular metabolism and integrate various cellular and environmental signals to enhance metabolism, with AKT having direct effects on glycolysis and glucose metabolism [20] and mTOR driving mitochondrial biogenesis via activation of PGC1α (Peroxisome proliferator‐activated receptor‐gamma coactivator‐1 alpha) and TFAM (mitochondrial transcription factor A) [21, 28, 29]. The suppressed metabolism in EPM1 iPSCs may simply result from the decreased anabolic demand due to the reduced proliferation, or it may be a protective response aimed at reducing ROS production. As antioxidant treatment normalised AKT/mTOR pathway activation and increased metabolic activity in patient cells, a protective response is a likely cause here.

CSTB has been suggested to regulate cell cycles [13, 14] and indeed the EPM1 patient iPSCs showed cell cycle defects. CSTB could control cell cycles through multiple pathways. Regulated protein degradation is critical for cell cycle progression and inhibition of critical proteases could lead to a delay in G1/S transition [30]. On the other hand, DNA damage response (DDR) is a crucial checkpoint in cell cycle regulation, and DDR can arrest cell cycle both in G1 and G2 stages, to allow time for repair before cell division [31]. In the EPM1 iPSCs both G1 and G2 phases were stalled, the cells harboured increased DNA damage and the MAPK/ERK pathway was activated, thus suggesting that DDR could be the cause of cell cycle stalling in these cells [32]. The increased oxidative stress is likely to be the cause of the DNA damage, although the CSTB defect can induce DNA damage also by other mechanisms, for example by reduced inhibition of histone cleaving nuclear cathepsin L and compromised chromatin structure [1].

The role for CSTB in regulating cell proliferation and apoptosis has become more evident in recent years when several groups have reported CSTB to play a role in cancer cell proliferation [13, 14, 15, 16, 33]. These studies suggest a direct role for CSTB in cell cycle regulation, likely through protein homeostasis. While strengthening the central role of CSTB in supporting cell survival and growth and showing that CSTB regulates these pathways also in EPM1, our data suggest oxidative stress as a central regulator for the proliferation defects in EPM1 cells. The relevance of these findings for EPM1 neuropathology and the role of oxidative stress in neurodegeneration in patients will need to be verified in a neuronal model. However, our results are in line with the finding that antioxidant treatment can alleviate neurological symptoms in some EPM1 patients [34, 35, 36], which supports oxidative stress as one of the main drives of EPM1 pathology [19, 37].

## 5 Conclusion

Downregulation of CSTB in EPM1 patients' pluripotent stem cells leads to increased lysosomal activity and oxidative stress, which increase DNA damage, drive cell cycle defects, suppress metabolism and increase apoptosis, thus reducing cell growth and survival.

## Author Contributions

S.S. designed and performed experiments, analysed data and wrote the original draft. L.P. performed experiments and analysed data. T.T. performed experiments. R.K. provided study material, revised the manuscript and supervised the study. R.H.H. designed and supervised the study, provided funding and wrote the manuscript. All authors have approved the final manuscript.

## Funding

This project was supported by the University of Eastern Finland, Biocenter Kuopio (Stem cell Center) and the European Union's Horizon 2020 research and innovation programme under the Marie Skłodowska‐Curie grant agreement grant number 101034307.

## Ethics Statement

Patient material was derived after informed written consent. All samples were kept pseudonymous throughout the study, and attention was paid to privacy, personal data protection and data management according to the principles of the EU GDPR (EU2016/679). Ethics Committee of KUH approved the study (410/2019).

## Conflicts of Interest

The authors declare no conflicts of interest.

## Supporting information


**FIGURE S1:** Plasmid maps. (A) pPB[shRNA]‐Neo‐U6 > hCSTB[shRNA#1] to silence CSTB expression (B) pPB[Exp]‐Neo‐EF1A > hCSTB[NM_000100.4] for overexpression of *CSTB*.
**FIGURE S2:** Characterisation of the Patient‐derived iPSCs. (A) Morphology of the patient iPS cells (scale bars 50 μm). (B) Immunocytochemistry for NANOG, TRA‐1–60 and TRA‐1–81 (scale bars 100 μm), DAPI for nuclear staining. *(C)* Expression of pluripotency associated genes, *DNMT3B*, *NANOG* and *SOX2*, by quantitative PCR. *(D)* Expression of Sendai virus mRNA by quantitative PCR (E) Karyotypes of the patient iPSCs.
**Table S1:** qPCR primer sequences.
**Table S2:** List of Antibodies.

## Data Availability

All data is available from the corresponding author upon reasonable request.
