# Supplementary material for: Oxidative Stress Drives Cell Cycle Stalling, Apoptosis and Metabolic Suppression in Cystatin B Deficient EPM1 Patient iPSCs
Source: Cell Prolif. 2026 May 19;59(10):e70232. doi: 10.1111/cpr.70232 (PMC13325810; doi:10.1111/cpr.70232)
Supplement: Supplementary file 1 [file CPR-59-e70232-s001.docx]

**Supplementary Information**

**Supplementary Figures**

**FIGURE S1**

**Plasmid maps.** (A) pPB[shRNA]-Neo-U6>hCSTB[shRNA#1] to silence CSTB expression (B) pPB[Exp]-Neo-EF1A>hCSTB[NM_000100.4] for overexpression of *CSTB*.

**FIGURE S2**

**Characterization of the Patient-derived iPSCs.** (A) Morphology of the patient iPS cells (scale bars 50 μm). (B) Immunocytochemistry for NANOG, TRA-1–60 and TRA-1–81 (scale bars 100 μm), DAPI for nuclear staining*. (C)* Expression of pluripotency associated genes, *DNMT3B*, *NANOG* and *SOX2*, by quantitative PCR*. (D)* Expression of Sendai virus mRNA by quantitative PCR (E) Karyotypes of the patient iPSCs.

**Supplementary Tables**

.

| *Gene* | Forward | Reverse |
| --- | --- | --- |
| *β-Actin* | TGTCCCCCAACTTGAGATGT | TGTGCACTTTTATTCAACTGGTC |
| *CDC25A* | TCTGGACAGCTCCTCTCGTCAT | ACTTCCAGGTGGAGACTCCTCT |
| *CDK1* | GGAAACCAGGAAGCCTAGCATC | GGATGATTCAGTGCCATTTTGCC |
| *CDK2* | ATGGATGCCTCTGCTCTCACTG | CCCGATGAGAATGGCAGAAAGC |
| *CSTB* | CGTGTCATTCAAGAGCCAGGTG | GCTTGGCTTTGTTGGTCTGGTAG |
| *CYCLIN D1* | TCTACACCGACAACTCCATCCG | TCTGGCATTTTGGAGAGGAAGTG |
| *CytC* | AAGGGAGGCAAGCACAAGACTG | CTCCATCAGTGTATCCTCTCCC |
| *DNMT3B* | GCTCACAGGGCCCGATACTT | GCAGTCCTGCAGCTCGAGTTTA |
| *LDHA* | AGCCCGATTCCGTTACCT | CACCAGCAACATTCATTCCA |
| *NANOG* | CCTGTGATTTGTGGGCCTG | GACAGTCTCCGTGTGAGGCAT |
| *ND6* | TGGTTGTCTTTGGATATACTACAGCG | CCAAGACCTCAACCCCTGAC |
| *SOX2* | GTATCAGGAGTTGTCAAGGCAGAG | TCCTAGTCTTAAAGAGGCAGCAAA |
| Sendai virus | GGATCACTAGGTGATATCGAGC | ACCAGACAAGAGTTTAAGAGATATGTATC |

**Table S1.** qPCR primer sequences

| Antibody Type | Antibody | Application/Dilution | Company Cat no. | RRID |
| --- | --- | --- | --- | --- |
| Primary Antibody | Mouse Anti-TRA-1–81 | IF (1:100) | Millipore; Cat# MAB4381 | AB_177638 |
|  | Rabbit Anti-Nanog | IF (1:300) | Abcam; Cat# ab21624 | AB_446437 |
|  | Mouse Anti-TRA-1-60 | IF (1:1000) | Chemicon; Cat# MAB4360 | AB_2119183 |
|  | Rabbit Anti-CSTB | WB (1:1000) | Sigma-Aldrich; Cat# HPA017380 | AB_1847414 |
|  | Rabbit Anti-Phospho-Histone H2A.X (Ser139) | IF (1:200) | Cell Signalling; Cat# 9718S | AB_2118009 |
|  | Rabbit Anti-Total Akt | WB (1:1000) | Cell Signalling; Cat# 4691S | AB_915783 |
|  | Rabbit Anti-Phospho-Akt (Ser473) | WB (1:1000) | Cell Signalling; Cat# 4075 | AB_916029 |
|  | Rabbit Anti-Total mTOR | WB (1:1000) | Cell Signalling; Cat# 2972 | AB_330978 |
|  | Rabbit Anti-Phospho-mTOR (Ser2448) | WB (1:1000) | Cell Signalling; Cat# 2971 | AB_330970 |
|  | Rabbit Anti-p44/42 MAPK (Erk1/2) | WB (1:2000) | Cell Signalling; Cat# 9102 | AB_330744 |
|  | Rabbit Anti-Phospho-p44/42 MAPK (Erk1/2) | WB (1:1000) | Cell Signalling; Cat# 4370 | AB_2315112 |
|  | Mouse Anti-β-Actin | WB (1:4000) | Bionordika; Cat# CABC004 |  |
|  | Mouse Anti-Total OXPHOS Human WB antibody | WB (1:1000) | Abcam; Cat# AB110411 | AB_2756818 |
| Secondary Antibody | Goat Anti-Rabbit IgG (HRP) | WB (1:3000) | VWR; Cat# IMMRIR2219 |  |
|  | Goat Anti-Mouse IgG (HRP) | WB (1:3000) | Jackson immuno; Cat# 115-035-003 |  |
|  | Chicken anti-Rabbit IgG (H+L) | IF (1:1000) | Thermo; A-21441 |  |

**Table S2.** List of Antibodies.
