# Supplementary material for: Oxidative Stress Drives Cell Cycle Stalling, Apoptosis and Metabolic Suppression in Cystatin B Deficient EPM1 Patient iPSCs
Source: Cell Prolif. 2026 May 19:e70232. Online ahead of print. doi: 10.1111/cpr.70232 (PMC13325810; doi:10.1111/cpr.70232)
Supplement: Supplementary file 1 — FIGURE S1: Plasmid maps. (A) pPB[shRNA]‐Neo‐U6 > hCSTB[shRNA#1] to silence CSTB expression (B) pPB[Exp]‐Neo‐EF1A > hCSTB[NM_000100.4] for overexpression of CSTB. FIGURE S2: Characterisation of the Patient‐derived iPSCs. (A) Morphology of the patient iPS cells (scale bars 50 μm). (B) Immunocytochemistry for NANOG, TRA‐1–60 and TRA‐1–81 (scale bars 100 μm), DAPI for nuclear staining. (C) Expression of pluripotency associated genes, DNMT3B, NANOG and SOX2, by quantitative PCR. (D) Expression of Sendai virus mRNA by quantitative PCR (E) Karyotypes of the patient iPSCs. Table S1: qPCR primer sequences. Table S2: List of Antibodies. [file CPR-9999-e70232-s001.docx]

**Supplementary Information**

**Supplementary Figures**

**
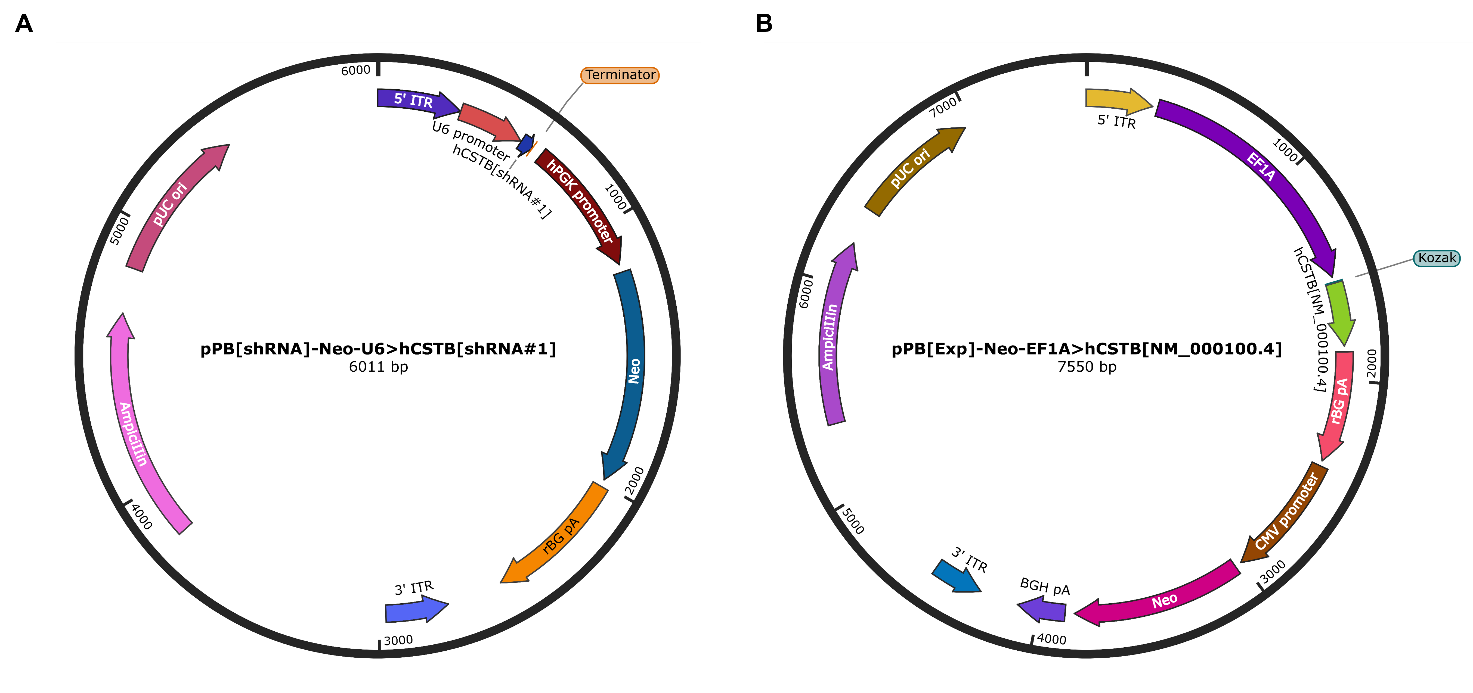
**

**FIGURE S1**

**Plasmid maps.** (A) pPB[shRNA]-Neo-U6>hCSTB[shRNA#1] to silence CSTB expression (B) pPB[Exp]-Neo-EF1A>hCSTB[NM_000100.4] for overexpression of *CSTB*.


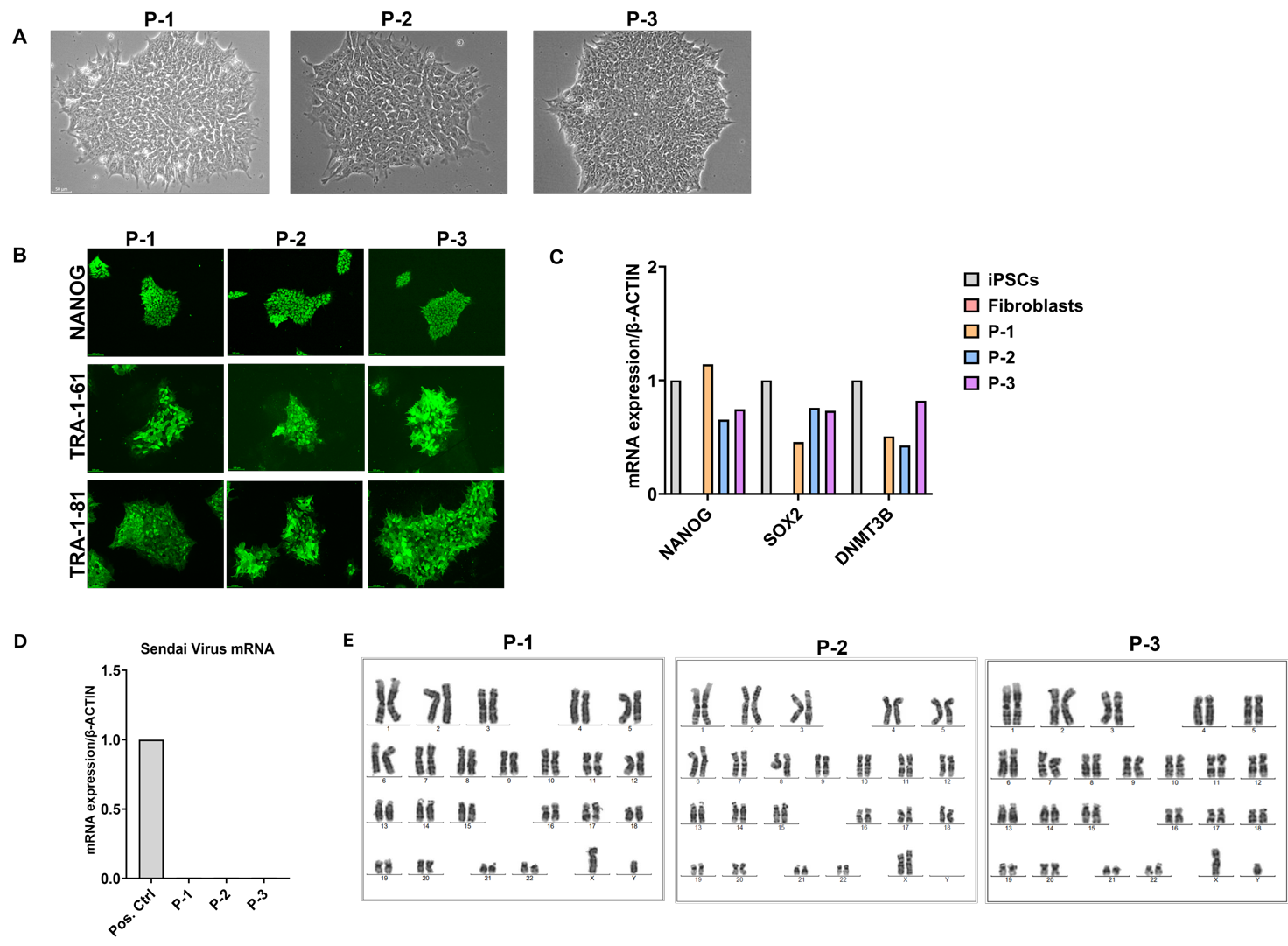


**FIGURE S2**

**Characterization of the Patient-derived iPSCs.** (A) Morphology of the patient iPS cells (scale bars 50 μm). (B) Immunocytochemistry for NANOG, TRA-1–60 and TRA-1–81 (scale bars 100 μm), DAPI for nuclear staining*. (C)* Expression of pluripotency associated genes, *DNMT3B*, *NANOG* and *SOX2*, by quantitative PCR*. (D)* Expression of Sendai virus mRNA by quantitative PCR (E) Karyotypes of the patient iPSCs.

**Supplementary Tables**

.

| *Gene* | Forward | Reverse |
| --- | --- | --- |
| *β-Actin* | TGTCCCCCAACTTGAGATGT | TGTGCACTTTTATTCAACTGGTC |
| *CDC25A* | TCTGGACAGCTCCTCTCGTCAT | ACTTCCAGGTGGAGACTCCTCT |
| *CDK1* | GGAAACCAGGAAGCCTAGCATC | GGATGATTCAGTGCCATTTTGCC |
| *CDK2* | ATGGATGCCTCTGCTCTCACTG | CCCGATGAGAATGGCAGAAAGC |
| *CSTB* | CGTGTCATTCAAGAGCCAGGTG | GCTTGGCTTTGTTGGTCTGGTAG |
| *CYCLIN D1* | TCTACACCGACAACTCCATCCG | TCTGGCATTTTGGAGAGGAAGTG |
| *CytC* | AAGGGAGGCAAGCACAAGACTG | CTCCATCAGTGTATCCTCTCCC |
| *DNMT3B* | GCTCACAGGGCCCGATACTT | GCAGTCCTGCAGCTCGAGTTTA |
| *LDHA* | AGCCCGATTCCGTTACCT | CACCAGCAACATTCATTCCA |
| *NANOG* | CCTGTGATTTGTGGGCCTG | GACAGTCTCCGTGTGAGGCAT |
| *ND6* | TGGTTGTCTTTGGATATACTACAGCG | CCAAGACCTCAACCCCTGAC |
| *SOX2* | GTATCAGGAGTTGTCAAGGCAGAG | TCCTAGTCTTAAAGAGGCAGCAAA |
| Sendai virus | GGATCACTAGGTGATATCGAGC | ACCAGACAAGAGTTTAAGAGATATGTATC |

**Table S1.** qPCR primer sequences

| Antibody Type | Antibody | Application/Dilution | Company Cat no. | RRID |
| --- | --- | --- | --- | --- |
| Primary Antibody | Mouse Anti-TRA-1–81 | IF (1:100) | Millipore; Cat# MAB4381 | AB_177638 |
|  | Rabbit Anti-Nanog | IF (1:300) | Abcam; Cat# ab21624 | AB_446437 |
|  | Mouse Anti-TRA-1-60 | IF (1:1000) | Chemicon; Cat# MAB4360 | AB_2119183 |
|  | Rabbit Anti-CSTB | WB (1:1000) | Sigma-Aldrich; Cat# HPA017380 | AB_1847414 |
|  | Rabbit Anti-Phospho-Histone H2A.X (Ser139) | IF (1:200) | Cell Signalling; Cat# 9718S | AB_2118009 |
|  | Rabbit Anti-Total Akt | WB (1:1000) | Cell Signalling; Cat# 4691S | AB_915783 |
|  | Rabbit Anti-Phospho-Akt (Ser473) | WB (1:1000) | Cell Signalling; Cat# 4075 | AB_916029 |
|  | Rabbit Anti-Total mTOR | WB (1:1000) | Cell Signalling; Cat# 2972 | AB_330978 |
|  | Rabbit Anti-Phospho-mTOR (Ser2448) | WB (1:1000) | Cell Signalling; Cat# 2971 | AB_330970 |
|  | Rabbit Anti-p44/42 MAPK (Erk1/2) | WB (1:2000) | Cell Signalling; Cat# 9102 | AB_330744 |
|  | Rabbit Anti-Phospho-p44/42 MAPK (Erk1/2) | WB (1:1000) | Cell Signalling; Cat# 4370 | AB_2315112 |
|  | Mouse Anti-β-Actin | WB (1:4000) | Bionordika; Cat# CABC004 |  |
|  | Mouse Anti-Total OXPHOS Human WB antibody | WB (1:1000) | Abcam; Cat# AB110411 | AB_2756818 |
| Secondary Antibody | Goat Anti-Rabbit IgG (HRP) | WB (1:3000) | VWR; Cat# IMMRIR2219 |  |
|  | Goat Anti-Mouse IgG (HRP) | WB (1:3000) | Jackson immuno; Cat# 115-035-003 |  |
|  | Chicken anti-Rabbit IgG (H+L) | IF (1:1000) | Thermo; A-21441 |  |

**Table S2.** List of Antibodies.
